# Examining the evolution and impact of OTC vending machines in Global Healthcare Systems

**DOI:** 10.1016/j.rcsop.2024.100540

**Published:** 2024-11-14

**Authors:** Ammar Abdulrahman Jairoun, Sabaa Saleh Al-Hemyari, Moyad Shahwan, Sahab Alkhoujah, Faris El-Dahiyat, Ammar Ali Saleh Jaber, Sa'ed H. Zyoud

**Affiliations:** a Health and Safety Department, Dubai Municipality, Dubai, United Arab Emirates; b Discipline of Clinical Pharmacy, School of Pharmaceutical Sciences, Universiti Sains Malaysia (USM), Pulau Pinang 11500, Malaysia; c Pharmacy Department, Emirates Health Services, Dubai, United Arab Emirates; d Centre of Medical and Bio-allied Health Sciences Research, Ajman University, Ajman, Ajman, 346, United Arab Emirates; e Department of Clinical Sciences, College of Pharmacy and Health Sciences, Ajman University, Ajman 346, United Arab Emirates; f Clinical Pharmacy Program, College of Pharmacy, Al Ain University, Al Ain, United Arab Emirates; g AAU Health and Biomedical Research Center, Al Ain University, Abu Dhabi, United Arab Emirates; h Department of Clinical Pharmacy & Pharmacotherapeutics, Dubai Pharmacy College for Girls, AlMuhaisanah 1, Al mizhar Dubai, United Arab Emirates; i Department of Clinical and Community Pharmacy, College of Medicine and Health Sciences, An-Najah National University, Nablus 44839, Palestine; j Clinical Research Centre, An-Najah National University Hospital, Nablus 44839, Palestine

**Keywords:** Over the counter, Nonprescription, Vending machine, Dispensing medicines, Automatic vendor

## Abstract

**Background:**

The study of over the counter (OTC) vending machines is crucial given their growing popularity and potential impact on the pharmaceutical industry and consumer behaviour.

**Objectives:**

This study involves a bibliometric quantitative analysis of academic literature to evaluate OTC vending machines in terms of their evolution, current trends, and potential areas for future research**.**

**Methods and materials:**

The Scopus database was searched using its advanced search tool, focusing on papers that included the search query in their titles, abstracts, and keywords. Data analysis included bibliometric indicators such as publication counts, citation trends, and co-authorship networks, which were visualized using VOSviewer software (version 1.6.20) to highlight key research themes and collaboration patterns.

**Results:**

A total of 399 publications on OTC vending machines were found between 1833 and 2024. Over the last 20 years, there has been an annual increase in the number of publications related to OTC vending machines, rising from 1 in 2001 to 31 in 2023. The United States (*n* = 118; 29.57 %) led in productivity, followed by the United Kingdom (45; 11.27 %), India (30; 7.51 %), Australia (27; 6.76 %), Canada (16; 4 %), Italy (15; 3.75 %), and China (15; 3.75 %). A total of 35 institutions have been involved in research on OTC vending machines. The Dubai Municipality contributed the highest percentage of articles (*n* = 3, 0.75 %), followed by the Emirates Health Services (n = 3, 0.75 %), Al Ain University (*n* = 2, 0.5 %), and Baystate Medical Center (n = 2, 0.5 %). Before 2016, much of the research on OTC vending machines focused on terms related to healthcare policy and health promotion, indicating the early exploration of this field. Present trends highlight terms associated with pharmacy practice, such as pharmacists, pharmacy, and prescription-related subjects.

**Conclusions:**

This study emphasises the practical necessity for enhanced regulatory structures to mitigate risks such as medication abuse, unfavourable drug interactions, and incorrect dispensing practices. Additionally, the study highlights the need for interdisciplinary collaboration among technologists, policymakers, and healthcare professionals to maximize the benefits of OTC vending machines while addressing consumer behaviour and safety issues.

## Highlights

• A review of OTC vending machines explored past trends, current state, and predicted future research hotspots.
• Vending machines enhance healthcare accessibility; academia-industry collaboration drives safer, user-friendly innovations.
• Ensuring safety and regulatory compliance is key to prevent misuse; policies must enforce strict rules and guidelines.

## 1 Introduction

The rising costs of healthcare and extended waiting times for doctors' appointments have led patients to seek alternative ways to manage their health. One increasingly popular solution involves purchasing non-prescription medications from non-pharmacy settings. However, this carries significant risks, including adverse events, exacerbating symptoms, and potentially worsening prognoses, particularly among chronically ill patients and the elderly.^1^ To enhance accessibility and convenience, these technology-driven devices contain medications and other pharmacy items like first aid kits and healthy food options, such as chocolate protein bars, energy drinks, and water detox products. They can be located in supermarkets, airports, malls, and schools, providing easy access to common non-prescription medications such as pain relievers (acetaminophen, ibuprofen, and aspirin), cold and flu medications, heartburn relievers, diarrhoea and constipation treatments, ointments, and creams.2., 3, 4. These machines allow patients to select products independently, enhancing privacy and convenience.

The use of OTC vending machines has increased globally, especially after the COVID-19 pandemic. They have been widely used in countries like the United States (USA), Canada, the United Kingdom, Switzerland, South Africa, and Norway. These machines are also used in some Arabic countries like the United Arab Emirates (UAE).^5^ However, in other countries like Ireland, non-prescription medications have not been made available to the public in vending machines and must be controlled by licensed pharmacists.^6^, ^7^, ^8^

OTC vending machines offer many benefits, providing convenient access to non-prescription drugs, reducing visits to pharmacies and clinics, and saving time for patients, especially during high-demand periods like pandemics. They are also cost-effective and provide a more private way to obtain medications, which is particularly valued by users.^4^^,^^7^^,^^8^, ^9^, ^10^, ^11^ Public perception tends to be positive, with many valuing the convenience and 24/7 access to medications.^4^ Indeed, a systematic review found that these automatic machines in community or outpatient pharmacies can reduce medication errors, increase pharmacist productivity, and save costs.^14^

However, these machines also present several challenges. The ease of access to medications can increase the risk of adverse effects, drug interactions, and misuse, particularly among elderly patients who are more likely to take multiple medications. The absence of professional guidance may lead to incorrect medication use, unnoticed side effects, and contraindications, potentially causing harm. The availability of medications like pseudoephedrine also raises concerns about addiction. Comprehensive studies are needed to address these gaps, particularly regarding safety, guidelines for use, and consumer behaviours and satisfaction.^4^^,^^12^^,^^13^ Therefore, regulatory compliance, security concerns, and measures to prevent access by children are critical issues that need to be considered to maximize the benefits of this innovative approach.^8^^,^^9^^,^^12^^,^^13^

OTC vending machines are being developed to be more easily controlled by the government and healthcare providers. They should include features like controlled access and authentication systems managed by the national health institutions, video consultations provided by healthcare professionals, and robust monitoring systems to enhance security. Additionally, secure payment options and proper medication storage conditions are essential for ensuring public safety and trust.^3^^,^^15^

The study of OTC vending machines is crucial given their growing popularity and potential impact on the pharmaceutical industry and consumer behaviour. A comprehensive examination is necessary to understand their effectiveness, safety, and influence on consumer behaviours. This research can provide insight for regulations, enhance machine design, and guide public health approaches to optimise advantages while reducing risks. Understanding how these machines affect consumer behaviour, and the pharmaceutical industry can lead to better integration into healthcare systems and more effective use of technology in providing healthcare services. Therefore, the objective of this study was to analyse the usage patterns, advantages, disadvantages, regulatory challenges, and consumer preferences associated with OTC vending machines.

## 2 Methods and materials

### 2.1 Database

This study employs bibliometric research, involving quantitative examination of scholarly literature to assess patterns of collaboration, impact, and trends within a specific topic. It examines the development and influence of research topics over time, utilising metrics such as co-authorship networks, publication counts, and citation analyses. This approach aids in identifying new research hotspots and provides insights into the progression of scientific knowledge. This study used the Scopus database to retrieve research on OTC vending machines due to its superior performance for bibliometric research.^16^^,^^17^ Scopus offers a broad range of peer-reviewed literature, including interdisciplinary studies, and provides reliable citation metrics, making it a preferred choice over databases like Web of Science, Medline, and Google Scholar.^18^, ^19^, ^20^, ^21^

Its comprehensive coverage and robust data quality make it ideal for analysing research trends across multiple disciplines. Its many advantages can be summarised as follows.

(1) Scopus not only makes a huge number of research studies available, but it covers multiple disciplines and types of literature, including articles in peer-reviewed journals, records of conference proceedings, trade publications, and other forms. Hence, it is the database of choice for analyses of research trends across more than one discipline.
(2) When selecting material for inclusion, Scopus applies systematic processes to ensure the data are of the necessary quality. It also has multiple content specialists who monitor and update data quality on an ongoing basis to maintain accuracy.
(3) Scopus facilitates complex searches by enabling multiple search criteria (e.g., author, publication, affiliation, keyword, and citation).
(4) Scopus provides the most frequently used citation metrics in bibliometric studies (citation counts, h-index, co-citation analysis, etc.) to evaluate publications and authors' impact and influence.
(5) Scopus enables the integration of other research aids like data visualisation tools and research analytics platforms, enabling researchers to more simply and clearly analyse and present bibliometric information.
(6) Scopus includes most of the journals indexed in its competitor databases and can thus be deemed a comprehensive resource for bibliometric analysis.

### 2.2 Search strategy

Keywords most likely to retrieve relevant studies from the Scopus online database on OTC vending machines were selected and input using the “advanced search” feature. Bias could be caused due to Scopus' practice of continually updating the database contents, so all the documents for this review were extracted and exported on march 29, 2024. Furthermore, the asterisk (*) was used as a wildcard and quotation marks (“”) were employed to locate specific terms or phrases within the studies searched. Having excluded errata and retracted documents from the search, a total of 399 articles were retrieved. The retrieved studies were then screened manually to ensure data quality. The complete texts were read by the researchers and the content of the articles was checked so that any irrelevant studies could be discarded. The search strategy was as follows

TITLE-ABS-KEY (("OTC" OR "Over the counter" OR "Non prescription*" OR "Medicine*" OR "Medication*" OR "Drug*" OR "Treat*" OR "Relieve*" OR "Pain Reliever*" OR "Acetaminophen" OR "Pain*" OR "Paracetamol" OR "analgesic*" OR "Ibuprofen" OR "Aspirin" OR "Antacid*" OR "Calcium carbonate tablets" OR "Famotidine" OR "H2 blocker" OR "Allergy Medications" OR "Loratadine" OR "Claritin" OR "Cetirizine" OR "Zyrtec" OR "Cold" OR "Cough" OR "Cough syrups" OR "Syrups*" OR "dextromethorphan" OR "Decongestant*" OR "Pseudoephedrine" OR "Antidiarrheal" OR "Loperamide" OR "Imodium" OR "Bismuth subsalicylate" OR "Pepto-Bismol" OR "Topical Analgesics" OR "Pain-relieving" OR "Cream*" OR "Patch*" OR "lidocaine patches" OR "Antifungal Creams" OR "Clotrimazole" OR "Miconazole" OR "Oral Rehydration solution*" OR "Electrolyte packets for rehydration" OR "Motion Sickness Medications" OR "Dimenhydrinate" OR "Dramamine" OR "Meclizine" OR "Bonine" OR "Nasal Sprays" OR "Sprays*" OR "Saline nasal sprays" OR "Nasal decongestant sprays" OR "Oxymetazoline" OR "Testing Kit*" OR "HIV Testing Kit" OR "Influenza Testing Kit" OR "COVID-19 Testing Kit") AND ("Vending machine" OR "Dispensing medicines" OR "Automatic vendor")) AND (LIMIT-TO (LANGUAGE "English"))

### 2.3 Validation of the search strategy

Any false positives returned by the search process outlined above were eliminated. The term “false positive results” in this study refers to items returned by the search query that were not explicitly related to OTC vending machines, often because of keyword overlap with unrelated topics. A manual screening process was performed to address this, examining titles, abstracts, and keywords to filter out unrelated papers. This improved the accuracy of the research findings by ensuring that only papers directly contributing to the study's focus were included. To this end, the 100 most-cited publications were identified among the extracted documents and analysed to ensure they did indeed address the topic of interest. Moreover, two bibliometric experts also checked the titles and abstracts of these 100 documents to ensure no false-positive results remained. After these checks to eliminate false positives, it was decided that this stage of the search was complete.

The ten most active scholars in this research area were also identified, and a correlation test was performed between their findings and the data retrieved by the search process to confirm the accuracy of the search strategy. The number of relevant publications identified by skilled bibliometric researchers through manual review and the number of relevant articles obtained by the advanced search query in the Scopus database were the two main factors compared. The purpose of this comparison was to ensure that the most relevant research on OTC vending machines was identified through an efficient search method.

The correlation coefficient can reveal the similarity in findings between retrieved publications and the real findings in the literature. It is also a means to locate false negatives, that is, publications or data that the initial search failed to identify, thus potentially indicating gaps or missed data in the initial query process. Researchers can therefore leverage the results of a correlation test to adjust the search strategy by, for example, changing keywords, search operators or fields, or exclusion/inclusion criteria to ensure their search is truly comprehensive.

The results of the correlation test indicated a correlation of *r* = 0.871, which is considered strong, and a statistically significant result (*p* < 0.001). Hence, the search query was accurate and valid, and the research findings are reliable and valid. The literature has many examples of this validation method being used in bibliometric reviews.^22^^,^^23^

As can be seen, a meticulous and comprehensive approach was taken to ensure the search query was accurate. The credibility of the findings is enhanced by both the participation of bibliometric experts and the findings of the correlation test. The current review and its findings are therefore believed to be of high quality and reliability.

### 2.4 Data export and data management (data analysis and visualisation)

After executing the search strategy, the next stage in the review process was to export the retrieved data into Microsoft Excel in “csv” format. For each document, exported data included titles, abstracts, authors' countries of origin, and institutions with which authors were affiliated. Moreover, the exported data included annual numbers of publications, types of documents, funding agencies, citations, and names of journals. In particular, the analyses conducted in this stage of the review focused on the percentages and frequencies of publication.

The VOSviewer software version 1.6.20 (Leiden University, Leiden, The Netherlands) was used to create network maps of terms extracted from the titles or abstracts of the included articles and of collaborations across countries. As one of the aims of this review was to predict future research hotspots, the same software program was employed to generate science-based knowledge networks revealing the progress of different research fields. VOSviewer enables users to carry out co-occurrence analysis to group terms into clusters that are then allocated unique colours for the purposes of identification. Hence, for the purpose of identifying research hotspots, it can be preferable to use cluster analysis rather than a co-occurrence network of terms in article titles and abstracts, as cluster analysis better reveals developing trends.

## 3 Results

#### 3.1 Overview of the retrieved publications

A total of 399 publications on OTC vending machines were found between 1833 and 2024, including 269 (74.18 %) articles, 29 (7.26 %) reviews, 13 (3.25 %) book chapters, and 61 (15.28 %) conference papers (Table 1).

**Table 1:** Types of Publications on OTC Vending Machines (*n* = 399).

Publication Type	Count	Percentages
Article	269	74.18 %
Book chapter	13	3.25 %
Conference paper	61	15.28 %
Conference review	4	1 %
Editorial	2	0.5 %
Letter	8	2 %
Note	10	2.5 %
Review	29	7.26 %
Short survey	3	0.75 %

#### 3.2 Exploring growth and productivity patterns

**Fig. 1:**
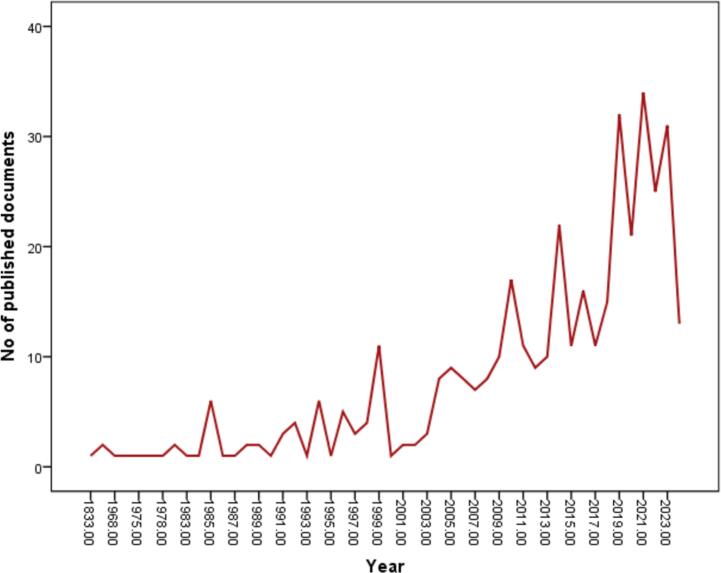
Trends in the growth of publications regarding the OTC vending machine from 1833 to 2024.

Over the last 20 years, there has been an annual increase in the number of publications related to OTC vending machines, rising from 1 in 2001 to 31 in 2023, as depicted in Fig. 1. The growth of these publications can be divided into two stages: an initial period (1833–1999) characterised by a slow production rate, followed by a later period (2000–2024) marked by significantly faster progress in publication output.

#### 3.3 Analysis of most prolific countries

Twenty-four countries participated in scientific research on OTC vending machines. The USA (*n* = 118; 29.57 %) led in productivity, followed by the United Kingdom (45; 11.27 %), India (30; 7.51 %), Australia (27; 6.76 %), Canada (16; 4 %), Italy (15; 3.75 %), and China (15; 3.75 %) (Table 2).

**Table 2:** Contributions of countries to scientific research on OTC vending machines.

Country	Documents (n)	%	Citations	Total link strength
USA	118	29.57	4223	27
United Kingdom	45	11.27	787	24
India	30	7.51	132	4
Australia	27	6.76	321	6
Canada	16	4.01	415	11
China	15	3.75	176	2
Italy	15	3.75	278	4
Japan	13	3.25	152	5
Germany	11	2.75	174	14
France	10	2.50	443	4
Malaysia	8	2.01	31	9
Poland	8	2.01	166	0
Spain	8	2.01	104	6
Netherlands	7	1.75	157	8
Thailand	7	1.75	72	8
UAE	7	1.75	18	9
Switzerland	6	1.50	109	7
Belgium	5	1.25	67	3
Denmark	5	1.25	52	1
Kenya	5	1.25	38	4
South Africa	5	1.25	48	11
South Korea	5	1.25	41	0
Sweden	5	1.25	81	4
Taiwan	5	1.25	10	1

**Fig. 2:**
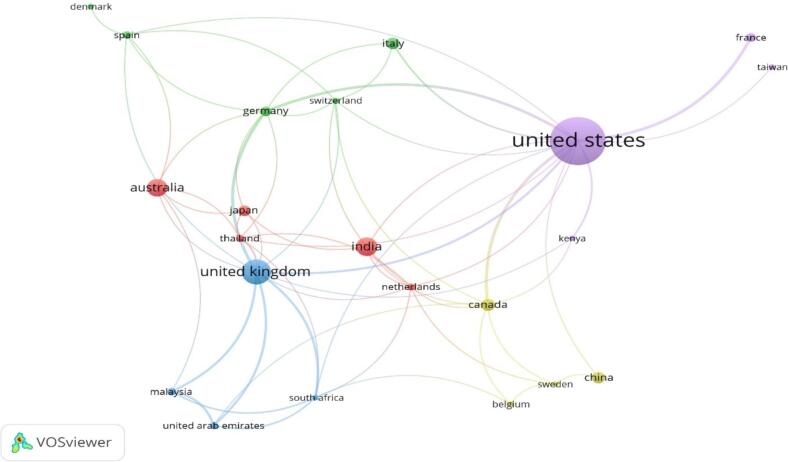
A visualisation map generated using VOS viewer software version 1.6.20, illustrating the international research collaboration network with a minimum contribution of 5 documents per country as the threshold (n = 24). Countries that are closely connected and have close relationships exhibit robust scientific collaboration. Conversely, countries on the periphery with weak connections to central countries demonstrate limited international research collaboration.

Fig. 2 illustrates a network visualisation map depicting research collaboration among these countries (*n* = 24) with a minimum contribution of five articles. The thickness of connecting lines and node size indicates the strength of cross-country collaboration, with the USA showing the most robust engagement.

Total link strength is the strength of the connections between various nodes (such as nations, organisations, or writers) in the co-authorship or collaboration network. A higher link strength indicates more frequent or stronger collaborations between entities.

#### 3.4 Analysis of most prolific institutions

A total of 35 institutions have been involved in research on OTC vending machines. The Dubai Municipality contributed the highest percentage of articles (*n* = 3, 0.75 %), followed by the Emirates Health Services (3, 0.75 %), Al Ain University (2, 0.5 %), and Baystate Medical Center (2, 0.5 %). The top ten institutions that published the most documents on OTC vending machines are detailed in Table 3.

**Table 3:** List of the top 10 institutions publishing research on OTC vending machines.

Institution	Country	n	%
Dubai Municipality	UAE	3	0.75
Emirates Health Services	UAE	3	0.75
Al Ain University	UAE	2	0.5
Baystate Medical Center, Springfield, Mass	USA	2	0.5
Stanford University School of Medicine	USA	2	0.5
Lebanese French University	Iraq	2	0.5
La Trobe University, Melbourne.	Australia	2	0.5
Centre of Medical and Bio-allied Health Sciences Research, Ajman University	UAE	2	0.5
College of Pharmacy and Health science, Ajman University.	UAE	2	0.5
University of Aix Marseille ii.	France	2	0.5

#### 3.5 Co-occurrence analysis

**Fig. 3:**
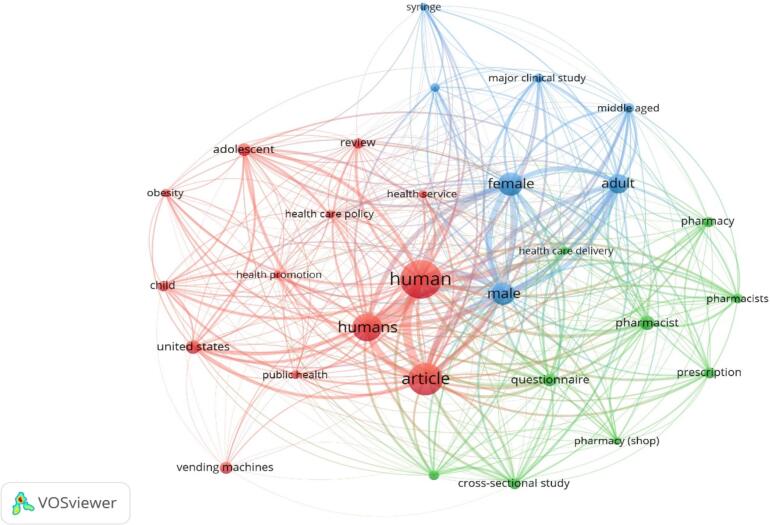
A cluster map was generated based on the analysis of terms found in titles or abstracts. The circle's size represents how often the terms appear, while various colours indicate different clusters. This map was created using VOSviewer software version 1.6.20.

The visualisation of frequently occurring terms in the titles and abstracts of collected documents, appearing at least 20 times, revealed 29 terms categorised into three coloured clusters (red, green, and blue) representing the top three research priority topics (see Fig. 3). The first cluster (red) encompassed terms concerning adolescents, child health, obesity, healthcare policies, health promotion, health services, and public health. The second cluster (blue) included terms related to demographics such as male, female, adults, middle age, human immunodeficiency, significant clinical studies, and syringe use. The third cluster (green) consisted of terms pertaining to pharmacy practice, including pharmacists, pharmacy, and prescription-related topics.

#### 3.6 Analysis of future research directions

**Fig. 4:**
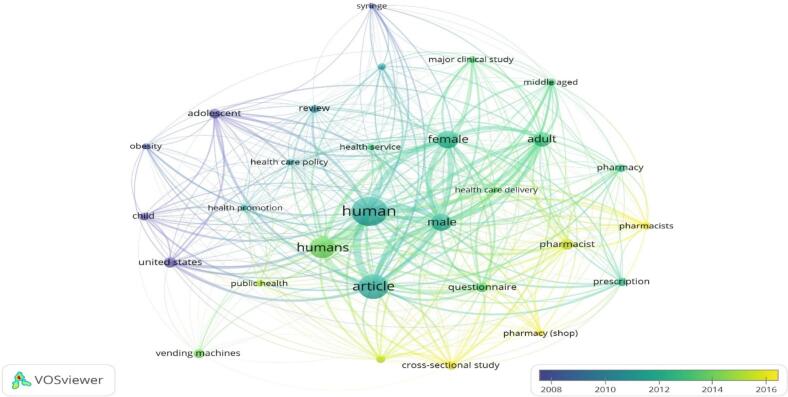
A network visualisation map generated to analyse the frequency of terms appearing in titles. Blue represents terms that appeared earlier, while yellow indicates later appearances. This map was created using VOSviewer software version 1.6.20.

Fig. 4 depicts an overlay visualisation where VOSviewer was used to colour terms based on their publication year. Initially, blue terms appeared, followed by yellow terms at a later stage. Before 2016, much of the research on OTC vending machines focused on terms related to healthcare policy and health promotion, indicating the early exploration of this field. Present trends highlight terms associated with pharmacy practice, such as pharmacists, pharmacy, and prescription-related subjects.

## 4 Discussion

This study demonstrates a growing interest in OTC vending machines, highlighting their potential to improve healthcare accessibility while emphasising the critical need for robust regulations and safety measures. The research aimed to assess usage patterns, benefits, disadvantages, regulatory challenges, and consumer preferences associated with OTC vending machines. The present study analysed a total of 399 publications. The diversity of publication types underscores the increasing academic and professional interest in this topic. Notably, there was a significant rise in publications over the past 20 years, from one in 2001 to 31 in 2023, illustrating the increasing appreciation for these machines in the healthcare industry, especially post-COVID-19.

The research involved 24 countries, with the USA leading with 118 publications, followed by the United Kingdom (45), India (30), Australia (27), Canada (16), Italy (15), and China (15). The collaboration network showed extensive cross-country partnerships, with the USA being particularly prominent. A total of 35 institutions contributed to this body of work, with notable contributions from Dubai Municipality and Emirates Health Services. This widespread international participation highlights a global interest in integrating OTC vending machines into healthcare systems.

Co-occurrence analysis of frequently occurring terms in the titles and abstracts revealed 29 terms categorised into three distinct groups: healthcare policies and public health, demographics and clinical studies, and pharmacy practice. This thematic classification highlights the complicated nature of the research, covering policy, clinical, and practical aspects of OTC vending machine usage.

A comparison with other research indicates a growing interest in vending machine technologies. While a systematic review in 2015 identified 136 publications on health product vending machines, focusing primarily on food options,^24^ this study's broader scope includes 399 publications, demonstrating a wider interest in including medications and other pharmacy products. Another study by Whatnall et al. (2020) identified 401 studies related to vending machines, aligning closely with previous findings.^25^ Another study analysed 98 articles on vending machines, with a higher number of publications appearing between 2014 and 2020. However, most of these studies were from English-speaking countries, such as the USA, the United Kingdom, Canada, and Australia, reflecting a strong research base in these regions.^26^The observed trends underscore the significance of vending machines, both medical and non-medical, as a viable solution for enhancing healthcare accessibility. Collaboration between academia and industry has the potential to advance technological innovations, minimising errors and improving user-friendliness. Safety and regulatory compliance were top research topics, appearing in 40 % of publications, indicating a critical need to address the several risks associated with these machines.^8^

The findings emphasise the importance of addressing safety issues and ensuring strict adherence to regulations to prevent misuse and potential negative outcomes. Policies should include stringent rules and guidelines for using these machines. In terms of their practical application, these machines should be integrated into healthcare systems to improve accessibility, especially in areas with limited healthcare access.^27^

Although OTC vending machines offer many benefits like reducing the number of pharmacy visits and offering privacy when obtaining medications, they also have several challenges that must be addressed. Safety concerns arise from their ease of misuse, incorrect use, and adverse drug interactions, especially among the elderly who are taking multiple medications. Ensuring regulatory compliance is crucial to control the types of medications dispensed and the security measures required to avoid abuse of certain drugs. Governmental and industry stakeholders must collaborate to develop strict regulations, establish secure systems for verifying identity and tracking usage, and educate the public on how to use them correctly. By addressing these issues, the benefits of OTC vending machines can be maximised while minimising associated risks.^28^

The recommendations in this study are derived from an extensive examination of publication trends, insights from significant research topics highlighted in the bibliometric analysis, and recurring themes identified through the co-occurrence network. This network revealed several focal clusters, including public health, pharmacy practice, and healthcare policies, indicating shifts in priorities within the field. Additionally, an analysis of publishing patterns underscored the growing need for robust regulatory frameworks, especially given the notable increase in studies related to safety and regulatory issues over the past 20 years.

These insights were synthesised to pinpoint practical areas for future research and policy development, such as the need for interdisciplinary collaborations to refine machine technology, promote consumer education to mitigate misuse risks, and enhance regulatory compliance for safe dispensing practices. These findings highlight the importance of integrating OTC vending machines within healthcare systems to maximize their positive impacts on public health outcomes and minimise potential adverse effects**.**

This bibliometric review of research into OTC vending machines analysed 399 research publications appearing between 1833 and 2024 and thus offers a robust and wide-ranging perspective on the research field. The study has several strengths, chief among them being the broad timespan considered and the extensive database used, both of which enable the detailed analysis of trends and evolution, providing valuable insights into future research possibilities. Moreover, by categorising publication types, the review indicates the multidisciplinary nature of the research field and underlines the global contributions made by certain countries and institutions at the forefront of collaborative research networks. Furthermore, the study's co-occurrence and temporal analyses shed light on key research themes and show how the research focus has changed over time.

There are several limitations to consider. Instead of assessing the rigor of each individual study, the focus was more on identifying publishing trends, co-authorship networks, and major research themes. Thus, a formal checklist was not employed to evaluate the methodological quality of the included studies. The absence of a rigorous quality evaluation may limit the interpretability of results from specific studies, which could impact the overall reliability of conclusions drawn from the dataset. Furthermore, the study was limited to papers indexed in the Scopus database, potentially excluding relevant studies from other databases, resulting in publication bias. A language bias may also exist as only English-language papers were reviewed, omitting significant studies published in other languages. Finally, because the co-occurrence network analysis focuses on frequently occurring phrases, it may overlook new or specialised study areas that are not yet prominent in the field. These limitations could be addressed in future research by expanding the range of databases used, implementing a rigorous quality evaluation, and broadening the analysis to include works in other languages and newly emerging fields of study.

## 5 Conclusion

This bibliometric analysis provides an extensive overview of the development of, and significant research on, over-the-counter vending machines. There has been a significant increase in publications over the last 20 years, indicating growing interest and use of these devices in global healthcare systems. There is a substantial focus on understanding the role of OTC vending machines in various healthcare contexts. The USA emerges as the principal contributor to research productivity. Predominantly, emerging research themes have concentrated on pharmacy practice and healthcare policy, highlighting the critical need to integrate these devices into clinical settings while ensuring their safe and regulated use. The results of this study emphasise the need for enhanced regulatory structures to mitigate risks such as medication abuse, unfavourable drug interactions, and incorrect dispensing practices. Additionally, the study highlights the need for interdisciplinary collaboration among technologists, policymakers, and healthcare professionals to maximize the benefits of OTC vending machines while addressing consumer behaviour and safety issues. Future research should focus more on these aspects, with an emphasis on developing regulations that balance patient safety and accessibility, and on advancing technology to ensure safe and effective integration into healthcare systems.

## Funding

No funding was used to assist in the preparation of this study.

## Ethical approval

The current study did not involve any interactions with humans, so approval from the Ethics Committee was not required.

## Ethics approval and consent to participate

Not applicable.

## CRediT authorship contribution statement

**Ammar Abdulrahman Jairoun:** Writing – review & editing, Writing – original draft, Visualization, Validation, Software, Methodology, Investigation, Formal analysis, Data curation, Conceptualization. **Sabaa Saleh Al-Hemyari:** Writing – review & editing, Writing – original draft, Resources, Methodology, Investigation, Data curation, Conceptualization. **Moyad Shahwan:** Writing – review & editing, Validation, Supervision, Resources, Project administration, Investigation, Funding acquisition, Conceptualization. **Sahab Alkhoujah:** Writing – review & editing, Writing – original draft, Resources. **Faris El-Dahiyat:** Writing – review & editing, Supervision, Project administration, Methodology, Funding acquisition, Data curation, Conceptualization. **Ammar Ali Saleh Jaber:** Writing – review & editing, Visualization, Software, Resources, Investigation, Data curation, Conceptualization. **Sa'ed H. Zyoud:** Writing – review & editing, Writing – original draft, Supervision, Software, Formal analysis, Data curation, Conceptualization.

## Declaration of competing interest

All authors declare that they have no conflict of interest.

## Data Availability

The original contributions presented in the study are available on request to the corresponding authors.
